# Simultaneous surgery for gastrostomy and laryngotracheal separation in a patient with Tay‒Sachs disease

**DOI:** 10.1038/s41439-024-00300-0

**Published:** 2024-11-29

**Authors:** Masaharu Moroto, Uda Daisuke, Tomoya Yodoi, Yoshihiro Nitta, Yohei Sugimoto, Tomohiro Chiyonobu, Hiroyuki Yamada, Kayo Ozaki, Taichi Nakatani, Norio Sakai

**Affiliations:** 1 Department of Pediatrics, Fukuchiyama City Hospital, Kyoto, Japan; 2 Department of Pediatrics, Maizuru Medical Center, Kyoto, Japan; 3 Department of Pediatrics, Kyoto Prefectural University of Medicine, Kyoto, Japan; 4 Department of Pediatrics, Kumihama Hospital, Kyoto, Japan; 5 Department of Molecular Diagnostics and Therapeutics, Graduate School of Medical Science, Kyoto Prefectural University of Medicine, Kyoto, Japan; 6 Department of Pediatrics, Toyooka Public Hospital, Hyogo, Japan; 7 Department of Endocrinology and Metabolism, Hyogo Prefectural Kobe Children’s Hospital, Hyogo, Japan; 8 Department of Pediatric Surgery, Hyogo Prefectural Kobe Children’s Hospital, Hyogo, Japan; 9 Center for Promoting Treatment of Intractable Disease, ISEIKAI International General Hospital, Osaka, Japan

**Keywords:** Paediatric neurological disorders, Disease genetics, Surgery, Paediatrics

## Abstract

Genetic testing identified novel compound heterozygous missense variants in the *HEXA* gene (NM_00520.6: c.775A>C and NM_000520.6: c.508C>T) in a 16-month-old girl diagnosed with Tay‒Sachs disease. The patient gradually became unable to consume food orally. She suffered severe aspiration pneumonia and underwent gastrostomy and laryngotracheal separation at 2 years and 4 months of age. Despite an initially good prognosis, she died at 3 years of age.

Tay‒Sachs disease (TSD, OMIM #272800) is caused by a loss of function of the enzyme β-hexosaminidase A (Hex A)^1^. TSD is an autosomal-recessive neurodegenerative lysosomal storage disorder^2,3^. The infantile form leads to death between 3 and 5 years of age. The cause of death is typically the primary disease, followed by aspiration pneumonia^4^. However, gastrostomy and laryngotracheal separation can prevent aspiration pneumonia in patients with chronic neuromuscular problems^5–7^.

The patient was born to nonconsanguineous parents at a gestational age of 39 weeks and 2 days (Fig. 1). No abnormalities were observed during the perinatal period. The patient passed the newborn hearing screening protocol, and newborn screening for inborn errors of metabolism revealed no abnormalities. Developmental stagnation was observed beginning at 8 months of age. At her 10-month health examination, the patient was referred to the nearest hospital. At 12 months, she could roll to only one side, and muscle hypotonia was apparent. Blood test results revealed elevated serum alanine aminotransferase (197 IU/L) and lactate dehydrogenase (814 IU/L) levels. Head MRI revealed no abnormal findings. G-banding revealed a 46,XX karyotype.

**Fig. 1:**
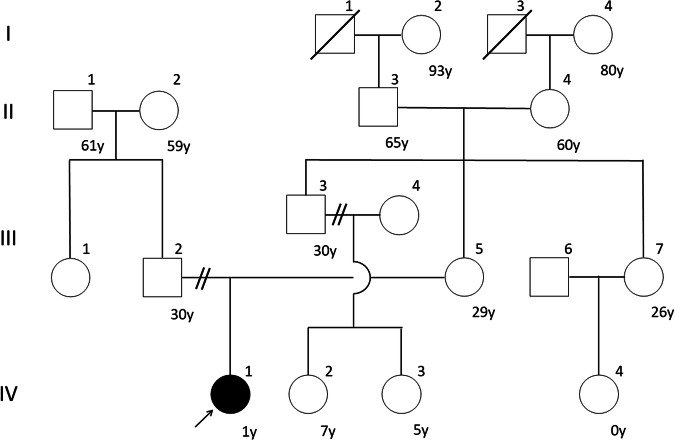
Family tree at diagnosis. The arrow indicates the index case. Squares indicate males, and circles indicate females. Upward diagonal stripes indicate deceased members. Black filled symbols indicate affected members, and white symbols indicate unaffected members. y years.

The underlying disease was unclear, so the patient was referred for consultation at our outpatient neurological clinic. No hepatosplenomegaly was observed. Although a small pericardial effusion was found, cardiac function was normal. We observed cherry-red spots in her eyes, and we subsequently tested for lipid storage diseases. While total hexosaminidase activity in leukocytes was maintained (1639.1 nmol/mg protein/h), Hex A enzyme activity decreased (18.1 nmol/mg protein/h). We diagnosed the patient with TSD at 16 months of age.

Genetic testing identified compound heterozygous missense variants in the *HEXA* gene. One missense variant (NM_000520.6: c.508C>T: p.(Arg170Trp)), inherited from her mother, was classified as pathogenic/likely pathogenic in ClinVar (https://www.ncbi.nlm.nih.gov/clinvar/). The other variant (NM_000520.6: c.775A>C: p.(Thr259Pro)), inherited from her father, was novel. The pathogenicity of this novel variant was evaluated according to the 2015 American College of Medical Genetics and Genomics guidelines^8^ and was classified as likely pathogenic (PM2 + PM3 + PP3 + PP4). The patient’s parents were divorced, so they did not plan to have another child. Her mother was concerned that the patient’s infant cousin might have the same disease (Fig. 1 IV-4), and genetic counseling was conducted. TSD is an autosomal-recessive disease, so there was a 50% possibility that the infant’s mother was a carrier; the infant’s father was nonconsanguineous. The incidence of carriers ranges from 1/277 in people of non-Jewish ethnicities^9^ to 1/1600 in East Asians^10^. After careful consideration, genetic testing was not performed. The infant had exhibited normal development at the time of assessment.

Although the patient received physical and speech therapy, her motor skill development was delayed. She was not able to move her limbs against gravity or to consume food or other substances orally. As a result, she became bedridden, and a nasogastric tube was inserted at 18 months of age. At 19 months of age, startle attacks, hyperacusis and versive seizures appeared. Electroencephalography revealed spike-and-wave discharges, so we diagnosed the patient with epilepsy and started a course of antiseizure medication. Her epilepsy was refractory, and levetiracetam was ineffective; as a result, she required two other antiseizure medications (valproate sodium and perampanel). Additionally, her mother reported that she exhibited a pale face and daytime apnea. Although vein blood gas analysis did not reveal an increase in carbon dioxide at the time of consultation, the continuous measurement of percutaneous oxygen saturation at home revealed that saturation occasionally decreased to less than 80% without the occurrence of seizures. Therefore, home oxygen therapy was introduced. When the patient was 2 years and 1 month old, she developed aspiration pneumonia. Noninvasive positive pressure ventilation was initiated, and a continuous saliva suction tube was introduced. However, she developed severe aspiration pneumonia 1 month later and was admitted to the intensive care unit. The patient underwent concurrent gastrostomy and laryngotracheal separation at 2 years and 4 months of age. An upper gastrointestinal examination before surgery revealed gastroesophageal reflux (GER), and a proton pump inhibitor was subsequently initiated. The patient’s muscles gradually became hypertonic, and clobazam was administered after the operation. Because the patient’s muscle tone weakened, she rapidly gained weight, and her enteral nutrition provision was adjusted. Although aspiration pneumonia was not observed, the patient was hospitalized multiple times because of respiratory infection and influenza. During hospitalization, respiratory suppression was observed, and tracheostomy-positive pressure ventilation (TPPV) was attempted. The patient’s breathing initially coordinated poorly with the ventilator. Her respiratory suppression gradually worsened, and nighttime TPPV was initiated at 2 years and 10 months of age. At 3 years of age, the patient was administered intravenous antibiotics to treat bronchitis for 6 days at the nearest hospital. Eight days after the patient improved, she died suddenly in the morning. An autopsy was conducted by the police, and the cause of death was determined to be heart failure, although the details were uncertain. Since the patient had GER, the vagal reflex may have been a possible cause of her sudden death (Fig. 2)^11,12^.

**Fig. 2:**
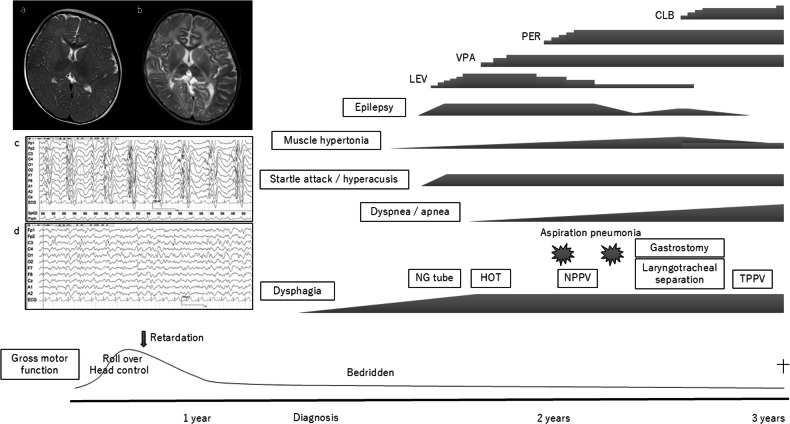
Clinical course. **a** T2-weighted brain MR image at 13 months, showing no abnormal findings. **b** T2-weighted brain MR image at 2 years and 4 months, showing cerebral atrophy and white matter hyperintensity. **c** Electroencephalogram at 19 months, showing generalized spikes and slow waves. **d** Electroencephalogram at 2 years and 3 months, with spikes dominantly appearing in the left central region. CLB clobazam, PER perampanel, VPA valproate sodium, LEV levetiracetam, NG tube nasogastric tube, HOT home oxygen therapy, NPPV noninvasive positive pressure ventilation, TPPV tracheostomy-positive pressure ventilation.

GM2 gangliosidosis is caused by a deficiency of one of the β-hexosaminidase lysosomal enzymes (Hex A, B, or S) or the GM2 activator protein (GM2A). The accumulation of GM2 ganglioside leads to neurodegeneration. TSD, caused by a deficiency of Hex A, occurs in one in 100,000 live births. Symptoms of the classical infantile form of TSD manifest before the age of 6 months, and TSD patients generally die by 3–5 years of age. However, some patients who survive beyond 5 years of age have been reported^4^. The cause of death is the primary disease, followed by aspiration pneumonia^4^.

Although various therapeutic strategies have been studied^13,14^, no specific therapeutic approach for TSD has been established. Bley et al. reported that hematopoietic stem cell transplantation (HSCT) could not prolong the lifespan of patients with the infantile form of TSD^4^. However, Prasad et al. reported that two of five children who underwent HSCT from the umbilical cord of an unrelated donor survived beyond 5 years of age^3,15^. Gene therapy with an adeno-associated virus vector is expected to show efficacy in TSD treatment, but it cannot be used in a clinical setting^16,17^.

The prevention of aspiration pneumonia is a major approach for extending the life of TSD patients. Laryngotracheal separation is a surgical procedure in which the upper respiratory tract is separated from the digestive tract. In two studies involving neurologically impaired children, Chida et al. and Shima et al. reported that patients who underwent tracheoesophageal diversion or laryngotracheal separation experienced a decreased number of hospitalizations and a decreased need for secretion suction^5,7^. Another approach for preventing aspiration is gastrostomy. Di Leo et al. evaluated children with neuromuscular disorders during long-term follow-up after gastrostomy. In patients who underwent gastrostomy, the reduction in respiratory infection was statistically significant^6^.

Our patient underwent simultaneous laryngotracheal separation and gastrostomy. Before the preoperative evaluation, the patient experienced GER, but she did not undergo fundoplication. For several months after the operation, the patient vomited frequently, but her vomiting gradually stopped. Thereafter, the time spent feeding the patient decreased. Her body weight markedly increased, so we reduced the number of calories provided and added trace elements. Although the prevention of aspiration pneumonia was achieved 8 months after the operation, the patient died unexpectedly. Considering the possibility of vagal reflex due to GER, fundoplication may be an approach to prevent sudden death. We conclude that concurrent laryngotracheal separation and gastrostomy was effective for prolonging the lifespan of this TSD patient, but further evidence is needed to reach a firm conclusion.

## HGV Database

The relevant data from this Data Report are hosted at the Human Genome Variation Database at 10.6084/m9.figshare.hgv.3462, 10.6084/m9.figshare.hgv.3465.
